# Therapeutic Potential of the Translation Inhibitor Silvestrol in Hepatocellular Cancer

**DOI:** 10.1371/journal.pone.0076136

**Published:** 2013-09-26

**Authors:** Takayuki Kogure, A. Douglas Kinghorn, Irene Yan, Brad Bolon, David M. Lucas, Michael R. Grever, Tushar Patel

**Affiliations:** 1 Department of Internal Medicine, College of Medicine, Ohio State University, Columbus, Ohio, United States of America; 2 Division of Medicinal Chemistry and Pharmacognosy, College of Pharmacy, Ohio State University, Columbus, Ohio, United States of America; 3 Comparative Pathology and Mouse Phenotyping Shared Resource, Ohio State University Comprehensive Cancer Center, Columbus, Ohio, United States of America; 4 Departments of Transplantation and Cancer Biology, Mayo Clinic, Jacksonville, Florida, United States of America; University College London, United Kingdom

## Abstract

**Background & Aims:**

Although hepatocellular cancers (HCC) frequently arise in the setting of fibrosis and a hepatic regenerative response requiring new cell growth, therapeutic strategies for these cancers have not targeted protein synthesis. Silvestrol, a rocaglate isolated from *Aglaia* *foveolata* , can inhibit protein synthesis by modulating the initiation of translation through the eukaryotic initiation factor 4A. In this study, we evaluated the therapeutic efficacy of silvestrol for HCC.

**Methods:**

The efficacy of silvestrol was examined using human HCC cells *in* *vitro* using an orthotopic tumor cell xenograft model in a fibrotic liver. The impact of silvestrol on the liver was assessed *in* *vivo* in wild-type mice.

**Results:**

Silvestrol inhibited cell growth with an IC50 of 12.5-86 nM in four different HCC cell lines. *In* *vitro*, silvestrol increased apoptosis and caspase 3/7 activity accompanied by loss of mitochondrial membrane potential and decreased expression of Mcl-1 and Bcl-xL. A synergistic effect was observed when silvestrol was combined with other therapeutic agents, with a dose-reduction index of 3.42-fold with sorafenib and 1.75-fold with rapamycin at a fractional effect of 0.5. *In* *vivo*, an antitumor effect was observed with 0.4 mg/kg silvestrol compared to controls after one week, and survival of tumor-bearing mice was improved with a median survival time of 42 and 28 days in the silvestrol and control groups, respectively. The effect on survival was not observed in orthotopic xenografts in non-fibrotic livers. Silvestrol treatment *in* *vivo* did not alter liver structure.

**Conclusions:**

These data identify silvestrol as a novel, structurally unique drug with potent anticancer activity for HCC and support the potential value of targeting initiation of translation in the treatment of HCC.

## Introduction

The high global incidence and mortality of hepatocellular cancer (HCC) emphasizes the need for therapies that are effective in improving survival [1]. HCC is highly refractory to conventional therapeutic approaches, and there is a need for more effective therapeutic agents to control these cancers. Cure is possible only with surgical resection or transplantation, but these are not possible for the majority of patients with this cancer, many of whom present with more advanced disease. Recent studies have implicated several diverse signaling mechanisms in the molecular pathogenesis of this cancer [2]. These account for the heterogeneity of responses and limit the utility of therapeutic interventions using conventional strategies that seek to modulate specific molecular targets [3,4]. At present only one agent, sorafenib, is available for systemic therapy with modest results in improving survival [5].

Tumor growth requires new protein synthesis and consequently is associated with an increase in protein translation. Directly targeting translation could be a useful therapeutic strategy, and warrants consideration for HCC [6,7]. Several studies have reported oncogenic effects arising from ectopic expression of the eukaryotic initiation factor eIF-4E, which is a rate limiting factor for translation inhibition [6]. Moreover, an anti-tumor effect of targeted down-regulation of eIF-4E has been shown in several studies using diverse tumor xenograft models. Targeting other components of the protein translation machinery have also been effective in modulating tumor cell growth. Modest antitumor efficacy for HCC has been shown using inhibitors of mammalian target of rapamycin (mTOR) pathway that are implicated in protein synthesis [8-10].

The rocaglate silvestrol is a cyclopenta[b]benzofuran flavagline from the *Aglaia* genus of the family Meliacae [11-13]. As a member of a novel class of drugs with a unique structure, silvestrol is an attractive compound to target HCC [14]. Silvestrol has the property of modulating translation by preventing ribosome loading onto mRNA templates by targeting the eukaryotic initiation factor, eIF-4A [15,16]. *In vivo* anti-tumor activity has been shown for silvestrol in hematological malignancies such as chronic lymphocytic leukemia, acute lymphocytic leukemia and mantle cell lymphoma [16-18], likely through the depletion of short half-life pro-growth or pro-survival proteins including cyclin D and Mcl-1. In animal studies using the Eµ-myc model, silvestrol can enhance sensitivity to standard agents such as doxorubicin [15]. Thus, we performed pre-clinical studies to evaluate the role of this unique compound for the treatment of HCC. Our data show that silvestrol is an effective cytostatic and cytotoxic agent for HCC cells both *in vitro* and in orthotopic tumor cell xenografts *in vivo*, and support further development of this agent as a therapeutic for HCC.

## Materials and Methods

### Ethics statement

All animal studies were performed following Institutional Animal Care and Use Committee procedures and guidelines at Ohio State University under an approved protocol.

### Cell Lines

Human HCC cell lines, PLC/PRF/5 (PLC), HepG2, Hep3B and Huh7 were obtained from the American Type Culture Collection (Manassas, VA) and were cultured in minimum essential medium with 10% fetal bovine serum (FBS) and 1% antimycotic/antibiotic mix. Luciferase-expressing PLC (PLC-luc), which were generated by stable transfection with luciferase-expressing phCMV plasmid containing cDNA encoding the ﬁreﬂy luciferase gene, were kindly provided by Dr Ching-Shih Chen (College of Pharmacy, Ohio State University, Columbus, OH). Cell line authentication was performed by Genetica DNA labs (Cincinnati, OH).

### Cytotoxicity Assay

Cell viability was assessed using the CellTiter 96 AQueous assay kit (Promega, Madison, WI). Cells (5,000/well) were plated in 96-well plates (BD Biosciences, Rockville, MD) and incubated at 37°C overnight prior to addition of experimental agents. Cell viability was assessed after 72 hours. IC_50_ values were calculated using XLfit software (IDBS, Burlington, MA). Drug-drug interactions were evaluated using a fixed ratio of concentrations. The results were analyzed using the median effects analysis and the combination index (CI) derived using the Calcusyn software program (Biosoft, Cambridge, United Kingdom).

### Apoptosis Assays

Cells were cultured in 4-well chamber slides (5 x 10^4^ cells per well) in 500 µL of medium and incubated at 37°C with 5% CO_2_. 100 nM of silvestrol was added and the extent of cell apoptosis was assessed after 24 hours. Cells with morphological changes of apoptotic cell death were quantitated using fluorescence microscopy after staining with 4', 6-diamidino-2-phenylindole (DAPI). For caspase-3/7 assays, cells were cultured in 96-well plates (5 x 10^3^ cells per well) and were 100 nM of silvestrol for up to 24 hours). Caspase-3/7 activity was assessed using a luminometric assay (Caspase-Glo 3/7 assay, Promega Corp., Madison, WI).

### Measurement of Mitochondrial Membrane Potential

Disruption of mitochondrial membrane potential was detected by using JC-1 (APO LOGIX™ JC-1, Peninsula Laboratories Inc., San Carlos, CA). For fluorescence microscopy, cells were cultured in a 4-well chamber slide (5 x 10^4^ cells per well) for fluorescence microscopy, or in 96-well plates (5 x 10^3^ cells per well) for fluorometric detection. Cells were incubated with 100 nM of silvestrol for 24 hours, then with 5 mg/mL of JC-1 at 37°C for 15 min. Intact mitochondria are detected as red aggregates with emission at 590 nm and mitochondrial membrane depolarization was detected as green fluorescence with emission at 530 nm.

### Western Blotting

Cells grown in 6-well plates were washed with phosphate-buffered saline (PBS) and lysed by incubation for 30 minutes in 600 µL of cell lysis buffer with protease inhibitors (Complete Lysis-M EDTA-free, Roche Diagnostics GmbH, Mannheim, Germany). Lysate protein concentrations were measured using a bicinchoninic acid assay kit (Protein Assay Kit, Pierce Biotechnology, Rockford, IL). ~25 mg of proteins were mixed with NuPAGE LDS Sample Buffer (Invitrogen, Carlsbad, CA) and proteins separated by sodium dodecyl sulfate-polyacrylamide gel electrophoresis (SDS-PAGE) using NuPAGE Novex 4-12% Bis-Tris Gels (Invitrogen). After electrophoresis, proteins on the gels were transferred to a polyvinylidene difluoride (PVDF) membrane (Bio-Rad Laboratories, Hercules, CA). The membranes were blocked with 5% bovine serum albumin (BSA) in Tris-buffered saline, and were incubated with primary antibodies and IRDye700- and IRDye800-labeled secondary antibodies (Rockland, Gilbertsville, PA) according to the manufacturer’s instructions. The protein of interest was visualized and quantitated using the LI-COR Odyssey Infrared Imaging System (LI-COR Bioscience, Lincoln, NE).

### Induction of hepatic fibrosis in nude mice

Six-week-old male imunodeficient mice (NCr nude) were obtained from Taconic (Hudson, NY) and fed food and water *ad libitum*. The mice were maintained in accordance with Institutional Animal Care and Use Committee procedures and guidelines under an approved protocol. Liver fibrosis was induced in nude mice by subcutaneous injection of 0.4g/kg of body weight carbon tetrachloride (CCl_4_) (Sigma-Aldrich, St. Louis, MO) twice weekly. CCl_4_ was diluted in olive oil (CCl_4_: olive oil = 1:7) and sterilized using 0.22 mm-filter prior to use. For validation studies to verify and quantitate liver fibrosis, CCl_4_ was administrated to nude mice twice weekly. After 6, 9, or 12 weeks, livers were extracted and fixed with formalin for pathological examination. After staining liver tissue with Masson’s trichrome, at least five randomly selected microscopic images of tissue sections from each mouse were photographed using digital imaging software (NIS-Elements, NIKON, Tokyo, Japan) and the percent area of liver fibrosis quantitated using NIH ImageJ software (National Institute of Health, Bethesda, MD).

### Orthotopic HCC cell xenograft model

CCl_4_ or diluent were administered subcutaneously twice weekly for 10 weeks as above, followed by direct intrahepatic injection of PLC-*luc* cells to establish orthotopic tumor cell xenografts in immunodeficient mice as follows. Laparotomy was performed using isoflurane anesthesia and the left liver lobe was exposed. PLC-luc cells (10^6^), were suspended in 20 mL of serum free medium containing 50% of Matrigel (BD Bioscience, San Jose, CA), and slowly injected into the left liver lobe using a 28-gauge needle. After the removal of the needle, compression was applied at the site of injection using a cotton swab to avoid bleeding. Abdominal wall muscle and skin were closed by continuous suture with absorbable suture material. Ten days after the tumor cell injection, bioluminescence imaging using the IVIS imaging system (Xenogen Corp., Alameda, CA) was initiated to monitor the establishment and growth of tumors. Mice were anesthetized with isoflurane and D-luciferin (Gold Biotechnology, St. Louis, MO) dissolved in PBS was administered intraperitoneally (150 mg/kg mouse body weight). After 10 minutes, mice were imaged with a highly sensitive, cooled CCD camera in a light-tight specimen chamber (IVIS200, Xenogen). Acquisition and quantiﬁcation of bioluminescence signals were performed using Living Image software (Xenogen). For a preliminarily study, five mice were sacrificed after at least four weeks of monitoring. Bioluminescence imaging was correlated with liver tumor volumes estimated using caliper measurements and a standard formula: 1/6*width*length*depth. For *in vitro* bioluminescence imaging, cells were counted and plated (40-10240 cells) on black 96-well plate. D-luciferin (150 µg/ml) was added to each well and imaging was performed after 10 minutes using IVIS.

### Treatment study in xenograft model

A treatment study was performed in orthotopic xenografts in nude mice with or without induction of hepatic fibrosis. After intrahepatic PLC-luc cell implantation, bioluminescence was monitored to monitor for development of tumors. Once bioluminescence intensity exceeded 10 million photon/sec, mice with orthotopic xenografts were randomized to receive silvestrol (0.4 or 1 mg/kg of body weight i.p) or control (0.5 mL/kg body weight 0.1% DMSO in saline) for 5 consecutive days a week for four weeks. A therapeutic response was defined as a > 30% reduction in bioluminescence intensity from baseline. Mice were imaged weekly for 10 weeks from the initiation of treatment.

### Hepatotoxicity investigation in wild-type mice

Six-week-old, female, C57BL/6 mice were treated with 0 (vehicle control) or 1.5 mg/kg of silvestrol (n = 7 per group) every 48 hr for 28 days by the intraperitoneal route. At necropsy, blood was collected to measure serum alanine (ALT) and aspartate (AST) aminotransferases, while the liver was fixed by immersion in neutral buffered 10% formalin. Histopathologic lesions in fixed, paraffin-embedded, hematoxylin and eosin (H&E)-stained tissue sections were graded by a board-certified veterinary pathologist using a 5-tiered, semi-quantitative scale: within normal limits, or minimal, mild, moderate, or marked changes.

### Chemicals and Reagents

Silvestrol, {6-O-demethyl-6-[6-(1,2-dihydroxyethyl)-3-methoxy-1,4-dioxan-2-yl]-aglafolin}, was isolated from the bark and twigs of Aglaia foveolata Pannell (Meliaceae), as described previously by A.D. Kinghorn. Silvestrol was resuspended in dimethylsulfoxide (DMSO) and stored at -80°C. Sorafenib was obtained from LC Laboratories (Woburn, MA) and diluted in DMSO. Rapamycin was obtained from Calbiochem (San Diego, CA) and diluted in DMSO. Concentration of DMSO were <0.1% for all studies. Z-VAD-FMK was obtained from Promega (Madison, WI)

### Statistical analysis

The data were analyzed by one-way analysis of variance (ANOVA) followed by a post hoc procedure. For the analysis of survival, survival curves were created by the Kaplan-Meier method and compared by a log-lank test. Multivariable analysis was performed with Cox proportional hazard regression. Statistical significance was accepted at a value of *p* < 0.05.

### Ethics statement

All animal studies were performed following Institutional Animal Care and Use Committee procedures and guidelines at Ohio State University under an approved protocol.

## Results

### Silvestrol inhibits human HCC cell growth *in vitro*

**Figure 1:**
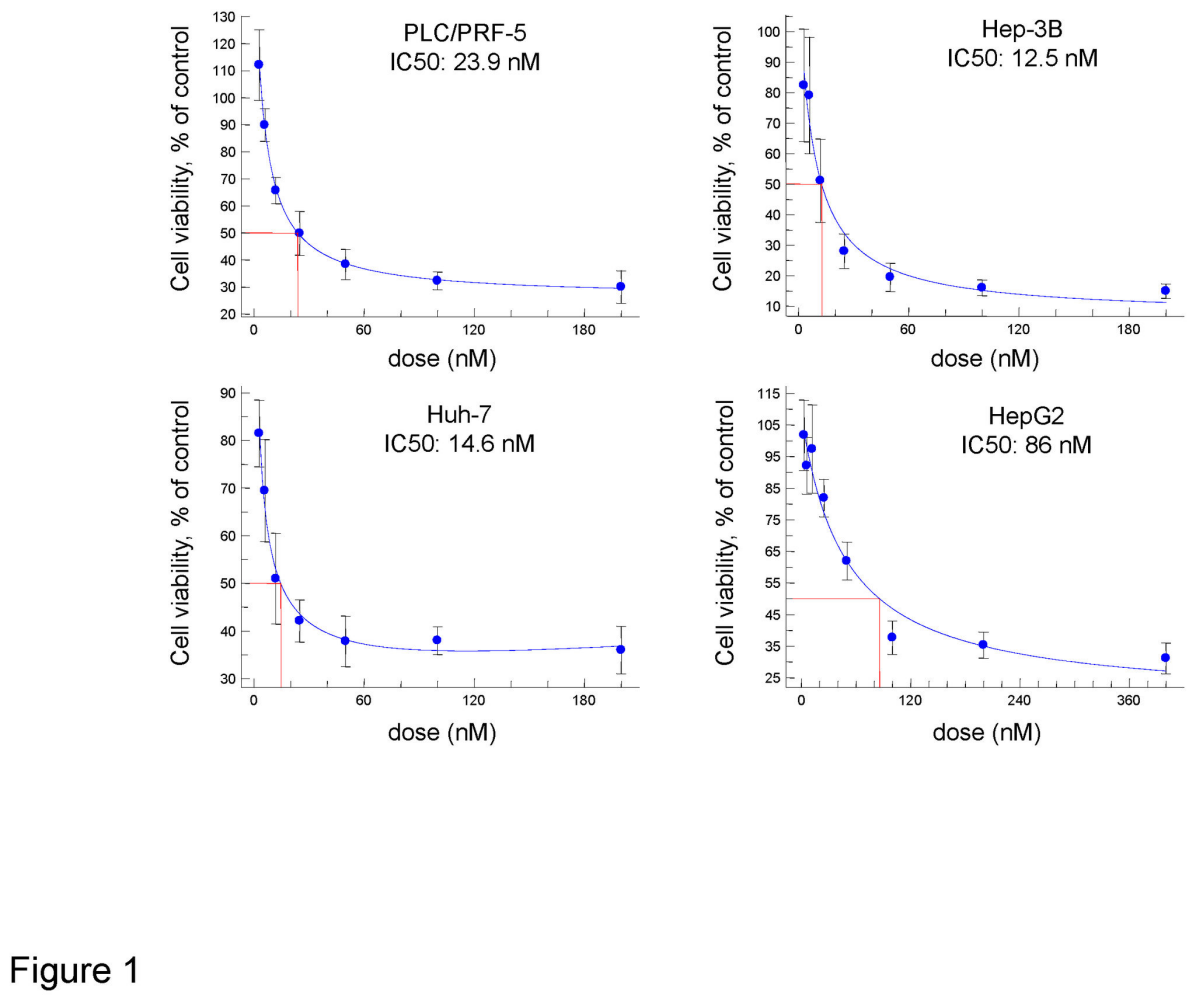
Cytotoxicity of silvestrol on HCC cells. Human HCC cells were seeded onto 96-well plates (5,000 cells/well) and incubated with various concentrations of silvestrol or control (diluent). Cell viability was assessed after 72 hours using a metabolic live cell assay (CellTiter 96 AQ, Promega Corp., Madison, WI). The data represents the mean and SD of five separate determinations.

To examine the potential anti-tumor effect of silvestrol, we began by examining the effect of silvestrol on cell growth *in vitro* using a panel of diverse malignant human hepatocyte cell lines. Incubation with silvestrol resulted in a concentration-dependent effect in inhibiting cell growth in all tumor cell lines examined, with an IC50 of 23.9 nM in PLC/PRF/5, 12.5 nM in Hep3B, 14.6 nM in Huh 7 and 86 nM in HepG2 cells (Figure 1). These data indicated that silvestrol had a highly potent anti-cancer effect in human HCC cells.

### Induction of cell death by apoptosis in HCC cells by silvestrol

**Figure 2:**
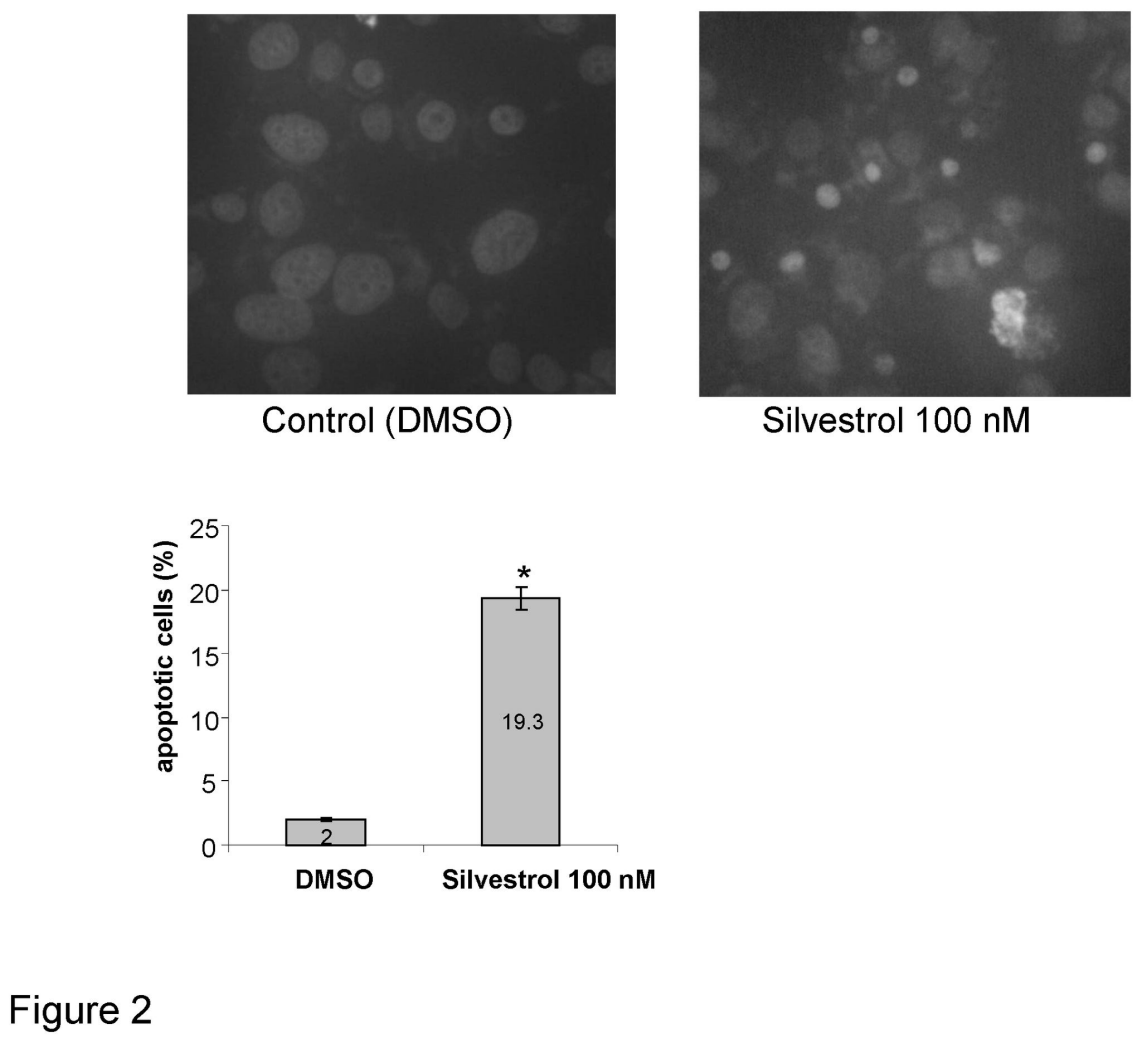
Induction of cell apoptosis by silvestrol. PLC/PRF/5 HCC cells were seeded onto 4-well chamber slide (25,000 cells/well) and were incubated with 100 nM silvestrol for 24 hours. Apoptotic cells were detected by fluorescence microscopy after DAPI staining. The proportion of cells showing morphological features of apoptosis were counted. *, *p* < 0.05, t-test.

We hypothesized that the cytotoxicity of silvestrol occurred as a result of induction of apoptosis mediated by impaired synthesis of short-lived apoptosis regulatory molecules such as Mcl-1 [16,17]. Thus, we examined the induction of apoptosis in PLC/PRF/5 human HCC cells following incubation with 100 nM silvestrol. Caspase 3/7 activation was detected by luminometric assay, with an increase in activity noted within 30 minutes and lasting for up to 6 hours (Figure 2A). Similarly, an increased cleavage of polyADP-ribose polymerase (PARP), a substrate for caspase activity, was also observed along with cleavage of other caspases such as caspase 8 and 9 (Figure 2B). Pre-incubation of cells for 30 minutes with the caspase inhibitor 50 µM zVAD-FMK reduced cytotoxicity from subsequent incubation with 50nM silvestrol from 36.4 ± 3.5% to 17.6 ± 2.9% after 24 hours. Consistent with these changes, 19.3% of PLC/PRF/5 cells showed morphological features of cells undergoing apoptosis after 24 hours (Figure 2C). These studies indicate that morphological and biochemical features of apoptosis occur early during incubation of HCC cells with silvetrol.

### Silvestrol alters expression of Mcl-1

**Figure 3:**
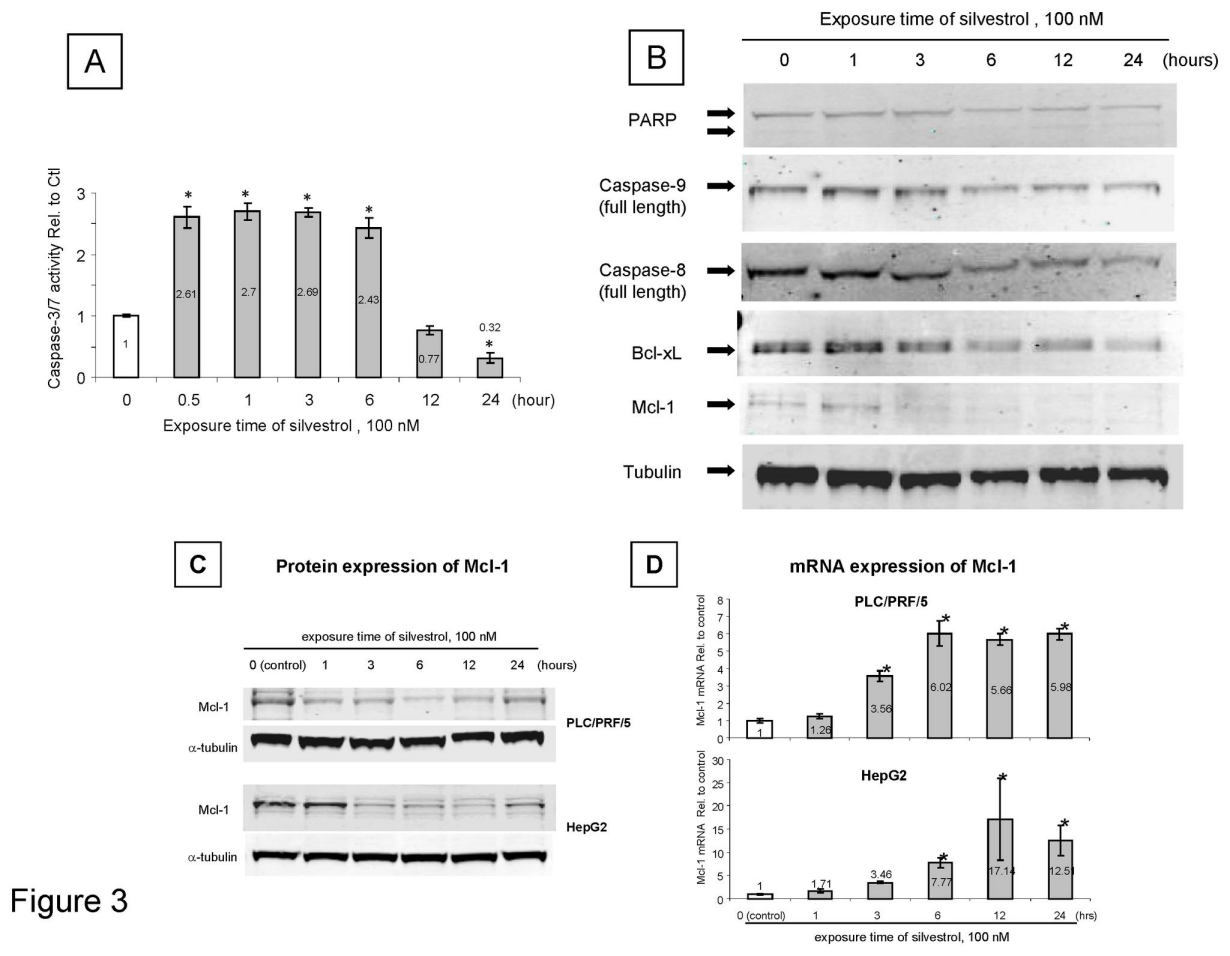
Modulation of expression of apoptosis-related proteins by silvestrol. (A) PLC/PRF/5 HCC cells were seeded on 96-well plate (5,000 cells/well) and incubated with 100 nM silvestrol for up to 24 hours. Caspase-3/7 activation was assessed using a luminometric assay (Caspase-Glo 3/7 Assay, Promega Corp., Madison WI). The data represents the mean and SD of three separate determinations. (B) PLC/PRF/5 cells were seeded on 6-well plates (20,000 cells/well) and incubated with 100 nM silvestrol (100 nM) for up to 24 hours. Cells were harvested at the indicated times, and immunoblot analysis for apoptosis-related proteins was performed. *, *p* < 0.05, ANOVA, Fisher’s PLSD (C) PLC/PRF/5 cells and HepG2 cells were seeded on 6-well plates and incubated with 100 nM silvestrol for up to 24 hours. Expression of Mcl-1 protein was assessed by immunoblotting. (D) RNA was extracted at each time point and Mcl-1 mRNA expression level was assessed by quantitative real-time PCR. Values are expressed relative to expression in no-treatment control after normalization using GAPDH as an internal control. The data represents the mean and SD. *, *p* < 0.05, ANOVA, Fisher’s PLSD.

**Figure 4:**
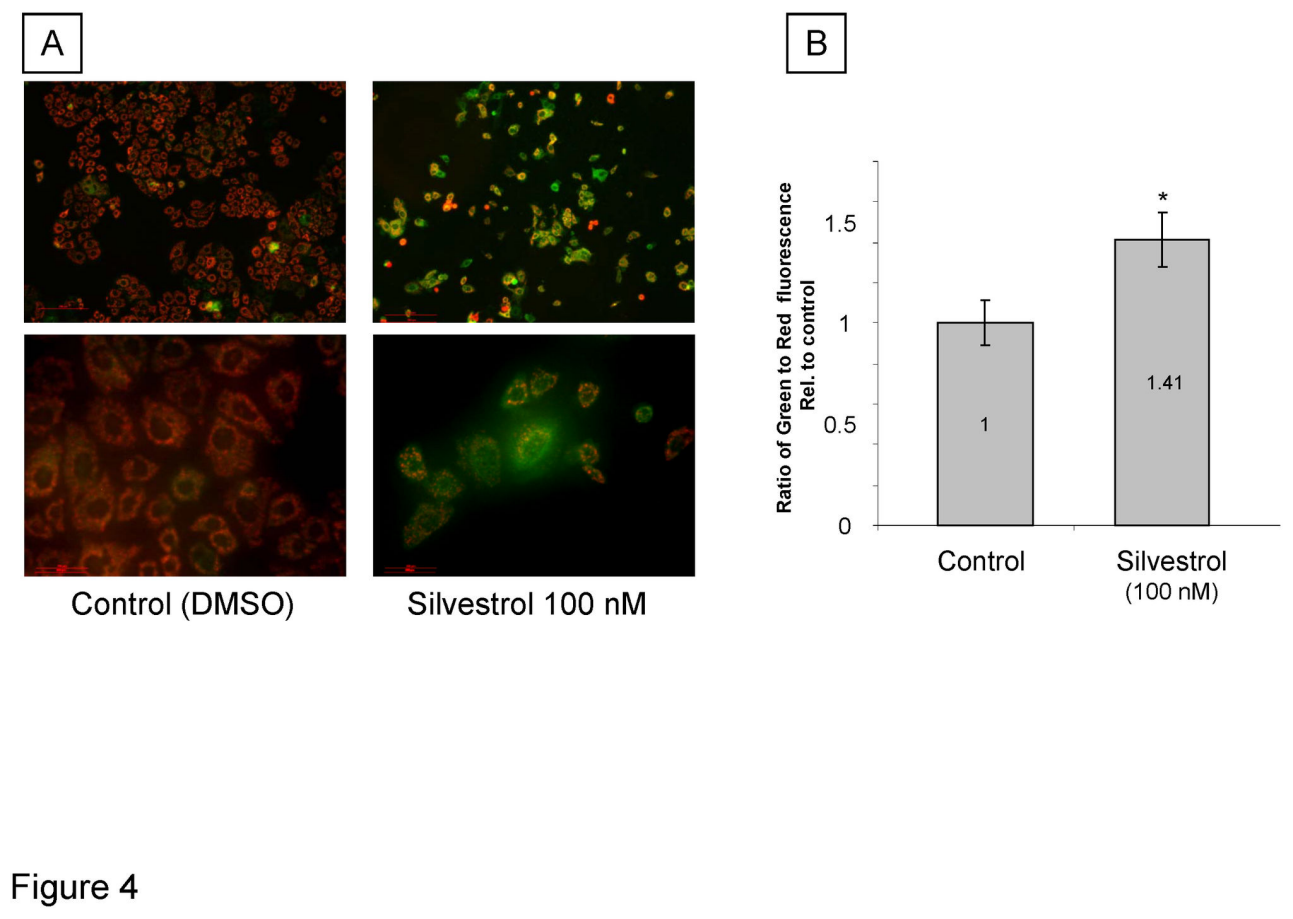
Decrease of mitochondrial membrane potential by silvestrol. A, PLC/PRF/5 cells were seeded on 4-well chamber slide (25,000 cells/well) and incubated with 100 nM silvestrol for 24 hours. Cells were stained with JC-1 and mitochondrial permeability was detected using fluorescence microscopy. Cells exhibit red fluorescence under basal conditions, but green fluorescence following alteration in mitochondrial membrane potential. B, PLC/PRF/5 cells were seeded on 96-well plate (5000 cells/well) and incubated with 100 nM silvestrol for 24 hours. Cells were stained with JC-1 and the ratio of green to red fluorescence was determined fluorometrically (mean ± SD). *, *p* < 0.05, t-test.

We postulated that the effect of silvestrol could be mediated by the impact on modulation of synthesis of short half-life regulators of apoptosis such as Mcl-1 and Bcl-X_L_. We therefore evaluated the effect of silvestrol on Mcl-1 protein and mRNA expression. A reduction in Mcl-1 protein expression lasting for up to 24 hours was noted in response to silvestrol in PLC/PRF/5 cells (Figure 3). In contrast, the expression of Mcl-1 mRNA was significantly increased. Similar changes were observed in HepG2 cells. As Mcl-1 can act as an anti-apoptotic factor to block the release of cytochrome C from mitochondria, we examined the effect of silvestrol on mitochondrial integrity. Incubation with 100 nM silvestrol for 24 hours resulted in a loss of mitochondrial membrane potential assessed by fluorescence microscopy after staining with JC-1 in PLC/PRF/5 cells (Figure 4A). JC-1 fluorescence ratio (red to green) increased to 141% of control in PLC/PRF/5 cells incubated with silvestrol (Figure 4B). Thus, the mechanistic effects of silvestrol on HCC cell growth could arise from enhanced apoptosis resulting from a decrease in mitochondrial membrane potential associated with a loss of Mcl-1 despite enhanced Mcl-1 mRNA transcription as described in CLL cells [17].

### Silvestrol modulates sensitivity to chemotherapy

**Figure 5:**
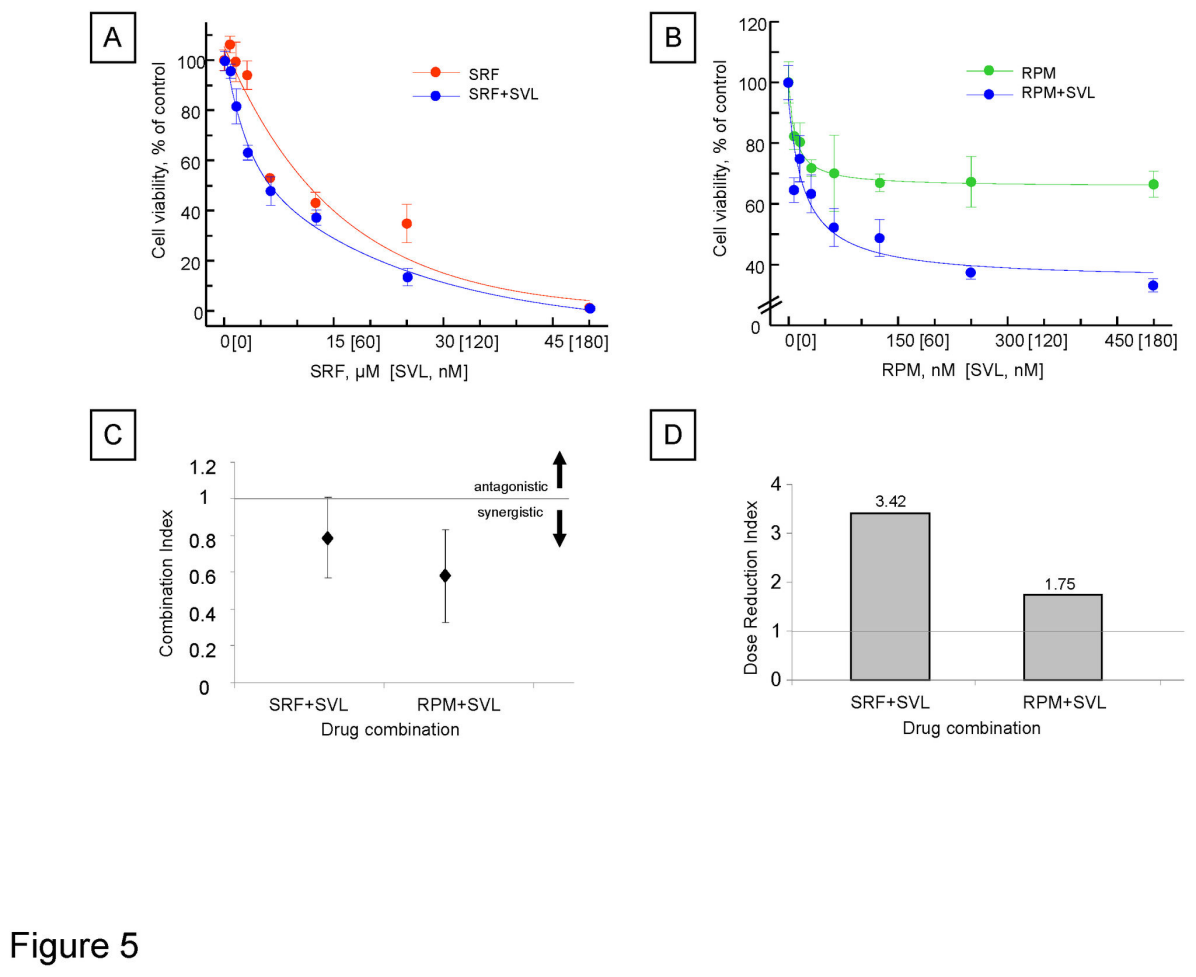
Synergistic effect between silvestrol (SVL) and sorafenib (SRF) or rapamycin (RPM). PLC/PRF/5 cells were seeded on 96-well plates (5000 cells/well) and were incubated for 72 hours with various concentrations of silvestrol (SVL) and (A) sorafenib (SRF) at a fixed ratio of SVL:SRF of 1:250 or (B) rapamycin (RPM) at a fixed ratio of SVL:RPM of 1:2.5. Cell viability was assessed using a metabolic assay (CellTiter 96 AQ, Promega Corp., Madison, WI). Potential interactions between silvestrol and sorafenib or rapamycin were evaluated using the median effects analysis of Chou and Talalay to derive the (C) combination index and (D) dose-reduction index of the combinations.

Given the intense interest in developing new therapeutic agents for HCC, it is likely that the clinical use of silvestrol could involve its use in combination with other therapeutic agents. Sorafenib is an oral multi-kinase inhibitor that has recently been evaluated and approved for use in advanced HCC. Although it has potent activity against the Raf/MEK/ERK pathway, the therapeutic efficacy for HCC may involve other pathways. mTOR signaling is frequently hyperactivated in HCC, and targeting mTOR is an attractive therapeutic strategy [19]. We examined interactions between silvestrol and either sorafenib or the mTOR inhibitor rapamycin. PLC/PRF/5 cells were treated with various concentrations of silvestrol in combination with sorafenib or rapamycin, and cytotoxicity was assessed after 72 hours. The combination of either sorafenib or rapamycin with silvestrol increased cell death induced by the latter (Figure 5A and 5B). Drug interaction evaluated by median effect analysis of Chou and Talalay were consistent with a synergistic interaction in growth inhibition with these combinations (Figure 5C) [20]. Furthermore, combination with silvestrol resulted in a favorable dose-reduction index for both sorafenib (3.42-fold reduction) and rapamycin (1.75-fold reduction) (Figure 5D). Thus, silvestrol can act synergistically with other chemotherapeutic agents that may be used for HCC.

### Murine orthotopic HCC xenograft model

**Figure 6:**
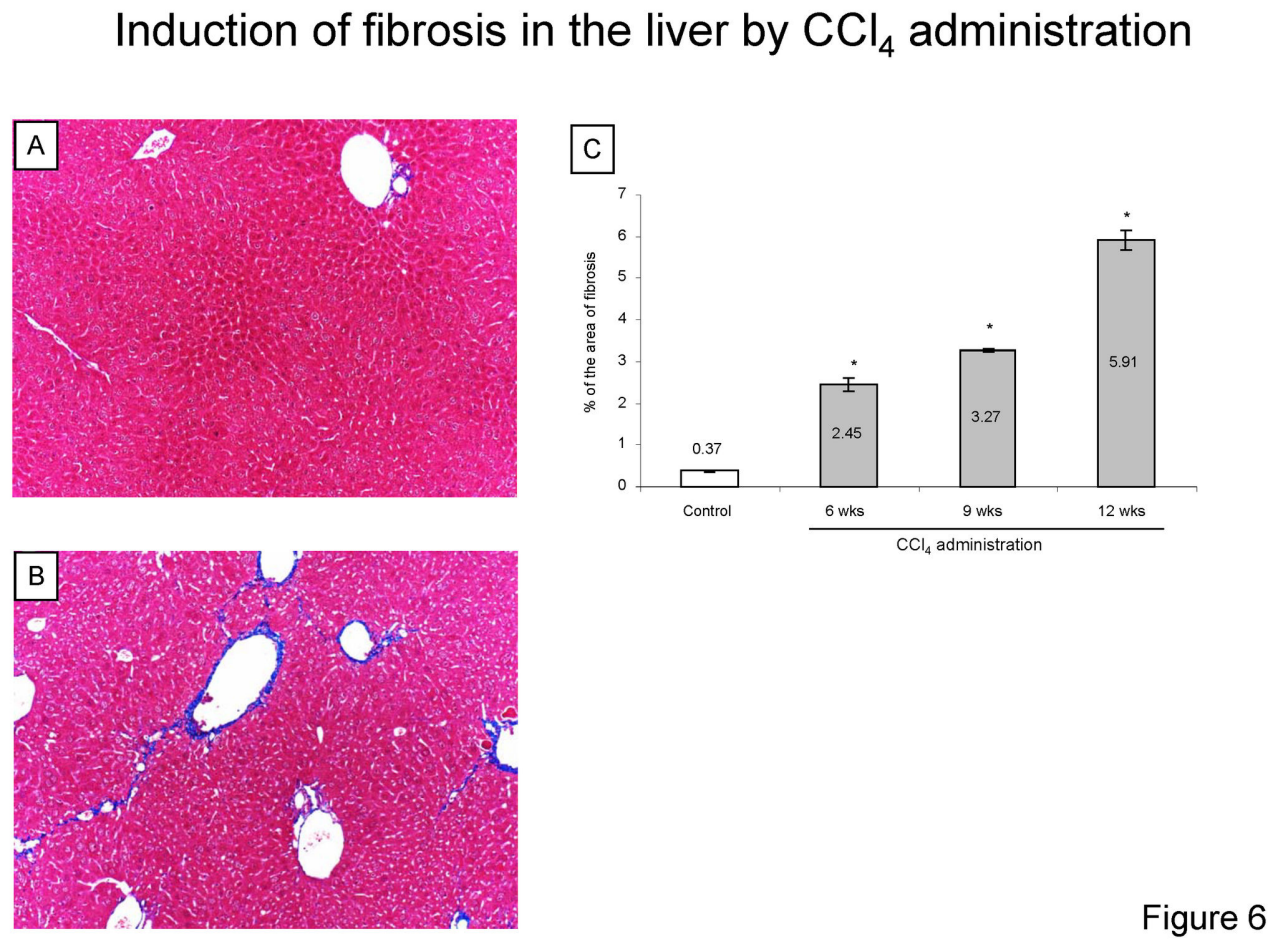
Induction of fibrosis in the liver by carbon tetrachloride administration. Hepatic fibrosis was induced in nude mice by twice weekly injection of carbon tetrachloride (CCl_4_) at the dosage of 0.4 g/kg body weight for 6, 9, and 12 weeks. *A* and *B*, typical pictures of liver histopathology with trichrome staining (A, control; B, CCl4 12 weeks). The liver was fixed with formalin and stained with Masson’s trichome. *C*, At least five images were randomly captured from each liver section and the percentage of the area of fibrosis was analyzed using NIH ImageJ software. Data represent the percentage of total area with fibrosis expressed as mean ± SD. *, *p* < 0.05, ANOVA, Fisher’s PLSD.

**Figure 7:**
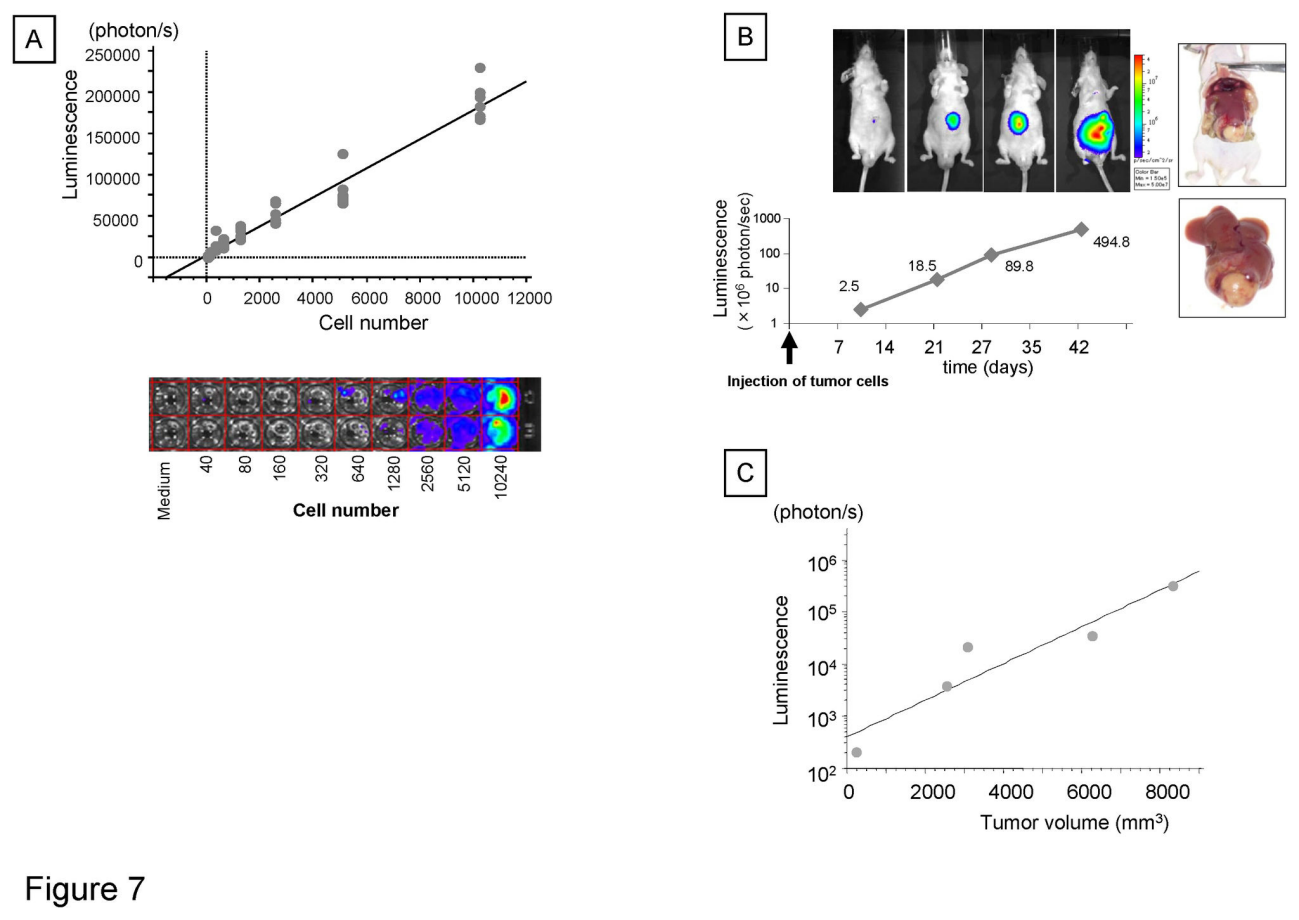
Estimation of tumor growth by bioluminescence. *A*, *In* *vitro* bioluminescence imaging of PLC-*luc* cells. Cells were counted and seeded on black 96-well plates. D-luciferin (150 µg/ml) was added to each well and imaging was performed using IVIS. Bioluminescence is plotted against cell number. *B*, *In* *vivo* bioluminescence imaging. Orthotopic tumors were established by direct intra-hepatic injection of PLC-*luc* cells. One million PLC-*luc* cells were injected into the left lobe of the liver. The growth of tumors were monitored by bioluminescent imaging using the IVIS. C, The relation between the tumor volume and bioluminescence. After at least 4 weeks of monitoring mice were sacrificed and liver tumor volumes were obtained using caliper measurement. The estimated volume of tumor after excision is plotted against bioluminescence determined in situ.

To evaluate the therapeutic use of silvestrol *in vivo*, we established a new disease relevant model of HCC by orthotopic tumor cell xenograft implantation in fibrotic or non-fibrotic livers in immunodeficient mice. The major advantage of this approach over conventional pre-clinical models such as subcutaneous tissue xenografts is that it more closely mimics the native tumor microenvironment. This enables assessment of disease-relevant microenvironmental influences on drug sensitivity *in vivo*. Liver fibrosis was experimentally induced by CCl_4_ administration, and the presence of fibrosis verified and quantitated by digital image analysis after Masson’s trichrome staining. Pathological examination showed typical fibrotic changes in the liver of mice with thickening of the portal tracts and bridging fibrosis (Figure 6A). The percentage of the area of fibrosis increased in dose of CCl_4_ dependent manner (Figure 6B). Next, PLC/PRF/5 cells constitutively expressing luciferase (PLC-*luc*) were established by stable transfection using a GFP-luciferase construct. The bioluminescence of these luciferase-expressing PCL/PRF/5 stable transfectants (PLC-luc cells) was verified *in vitro*, with a strong positive correlation observed between bioluminescence intensity and the number of cells *in vitro* (Figure 7A) (*r* ^2^ = 0.973). Next, orthotopic xenografts were established by intrahepatic injection of PLC-luc cells (10^6^ cells) into the left liver lobe of nude mice (8-week old, male, n = 5) and bioluminescence imaging performed to monitor the establishment and growth of the liver tumor starting at 10 days after the implantation (Figure 7B). The bioluminescence within the liver was noted to increase with time. After at least 4 weeks, mice were sacrificed and the volume of extracted tumors was obtained by caliper measurement. There was a positive correlation between the bioluminescence intensity prior to liver tumor extraction in vivo, and tumor volume (*r* ^2^ = 0.638) (Figure 7C). These studies validated the use of the PLC-luc cells and bioluminescence imaging to measure tumor growth *in vivo*.

### Liver fibrosis modulates tumor cell growth

**Figure 8:**
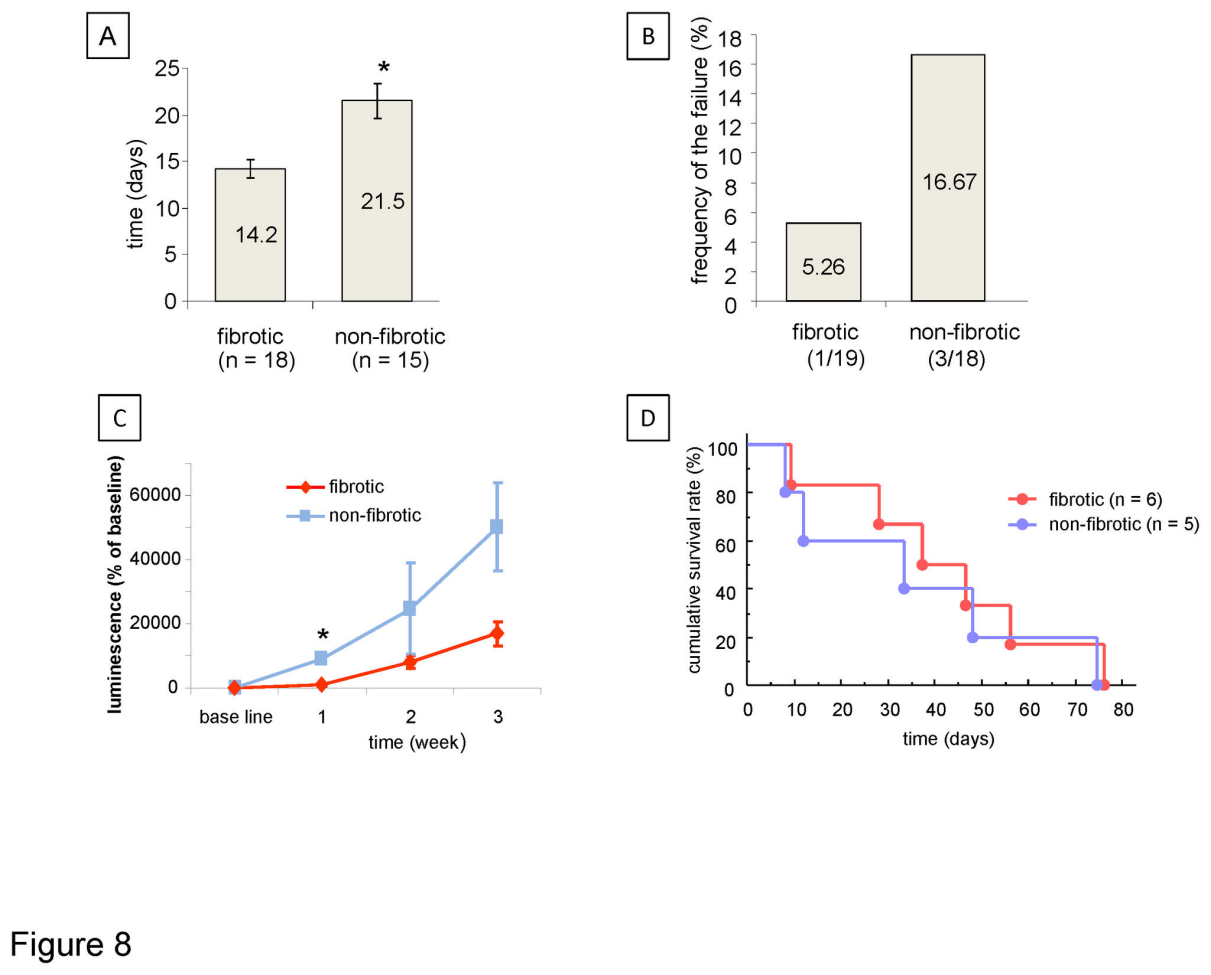
Effect of liver fibrosis on tumor initiation and growth. Mice received 0.4 g/kg carbon tetrachloride to induce liver fibrosis (n=19) or vehicle injections (n=18) for ten weeks. Orthotopic tumors were then established by direct intra-hepatic injection of 10^6^ PLC-*luc* cells. Tumor cell xenograft growth was monitored by bioluminescence imaging and was detected in 18 mice with fibrotic livers and in 15 mice without liver fibrosis. (A) The time in days (mean ± SE) for liver tumor growth from tumor cell injection to bioluminescence intensity of 10^7^ photon/sec is shown. (B) Frequency of failure of tumor formation (%). (C) Effect of fibrosis on tumor growth. The change in bioluminescence intensity as a percent of baseline (mean ± SE) is plotted against time. Once bioluminescence exceeded 10^7^ photon/sec, each mouse was randomized to a treatment arm and receive silvestrol or diluent (control). D, Survival curve of *control* group. The presence of liver fibrosis did not affect the survival of mice with tumor xenografts that did not receive any treatment. *, *p* < 0.05.

The presence of liver fibrosis is a major determinant of HCC development (Figure 8). We first investigated the effects of fibrosis on tumor initiation and tumor growth. Nineteen mice each were subcutaneously administered 0.4 mg/kg of body weight of either CCl_4_ (19 mice) or vehicle (18 mice) twice weekly for 10 weeks. Intrahepatic tumor cell implantation was performed after 10 weeks, at which time fibrosis is established in all mice receiving CCl_4_. One mice in the vehicle group died immediately following tumor cell implantation surgery. Four mice, one in the CCl_4_ group and three in vehicle treated group, did not develop liver tumor after at least 12 weeks following implantation. One CCl_4_ treated mouse that developed measurable tumor died before starting treatment. The remaining 32 liver tumor bearing mice comprised of 17 CCl_4_ treated mice (fibrotic liver group), and 15 vehicle treated mice (non-fibrotic liver). Once tumor formation was detected, with bioluminescence intensity >10 million photon/sec, each mouse in each group (fibrotic/ non-fibrotic liver) was randomized to receive either silvestrol (0.5 mg/kg or 1 mg/kg body weight) or DMSO 0.1% (control) by i.p injection five consecutive days a week for four weeks. One mouse was randomized but died before receiving any injections and was not included in the analysis. The mean duration from tumor cell injection to randomization was significantly decreased in mice with hepatic fibrosis compared to non-fibrotic mice (14.2 days *vs* 21.5 days, p = 0.0011, (Figure 8A). The frequency of failure of tumor development was lower in fibrotic mice (1 of 19 mice, 5.26%) than in non-fibrotic mice (3 of 18 mice, 16.7%) (Figure 8B). There was no statistically significant difference in cumulative survival between non-fibrotic mice and fibrotic mice in the vehicle-treated control group (p=0.59), and the median survival times calculated using the Kaplan-Meier method were 25 days for non-fibrotic mice, and 28 days for fibrotic mice.

### Silvestrol does not damage normal hepatocytes *in vivo*

Silvestrol administration at 1.5 mg/kg every other day for 28 days did not induce visible lesions in the hepatic parenchyma of any mouse. In particular, neither necrosis nor apoptosis were evident within hepatocytes, bile duct epithelium, or resident sinusoidal macrophages (i.e., Kupffer cells). Relative to control animals, treatment with silvestrol was associated with modest elevations in the serum activities of ALT and AST in 3 of 7 mice. The inconsistent occurrence coupled with the minimal (n = 2) or mild (n = 1) extent indicate that these enzymes will not be useful as standalone biomarkers for silvestrol exposure.

### Silvestrol improves survival from tumor growth *in vivo*

**Figure 9:**
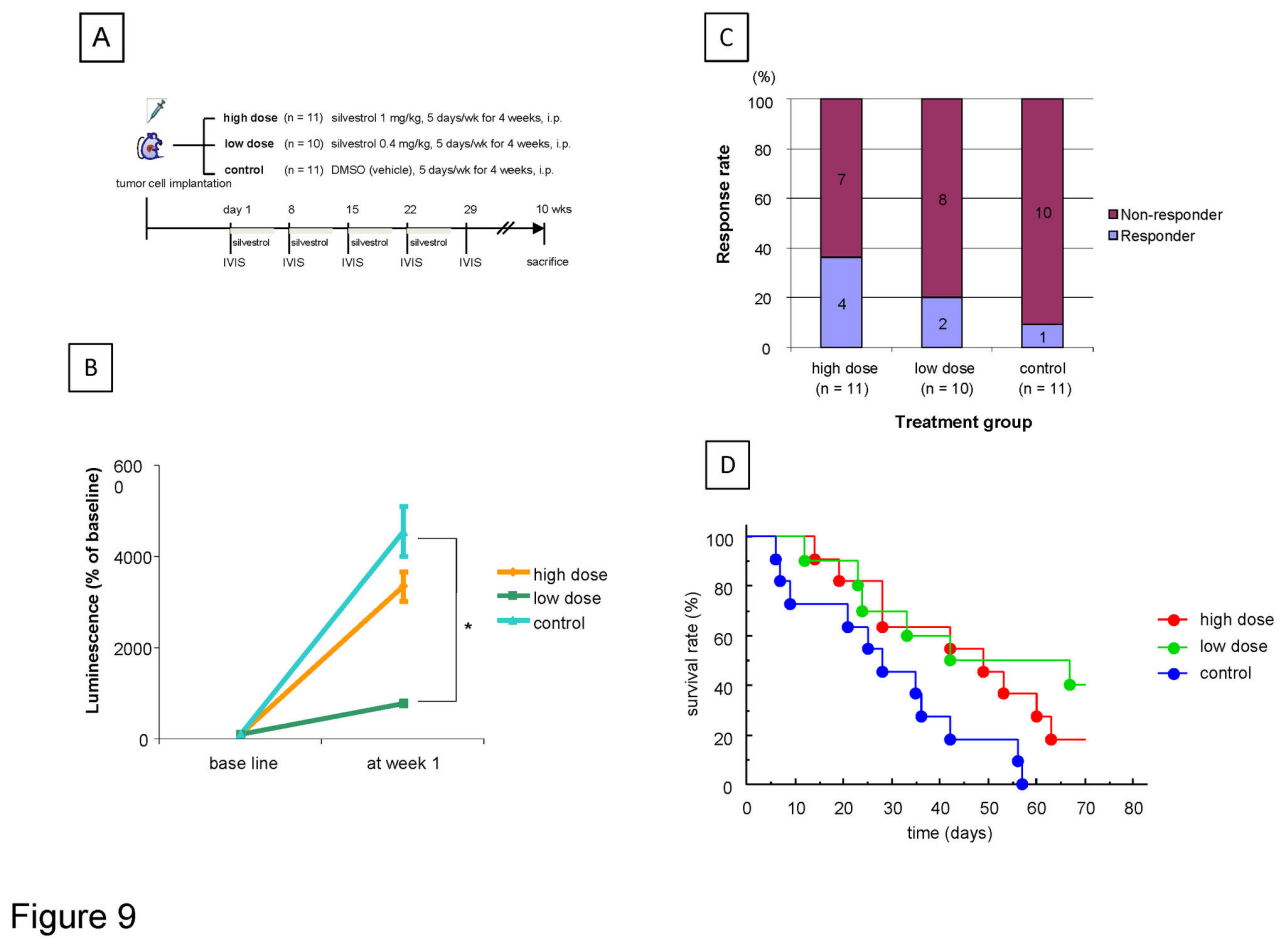
Anti-tumor effect of silvestrol in orthotopic human HCC xenografts in nude mice. A. Treatment protocol. B. Luminescence (% of baseline) at one week after the treatment in each group (mean ± SE) are shown. *, *p* < 0.05. C. Response rate in each treatment group is shown. The percentage of mice with a response defined as reduction in bioluminescence of >30% is shown. D. Survival curves of each treatment group by Kaplan-Meier method are shown. * control vs. high dose p=0.037. ** control vs. low dose, p=0.024.

Initial response was determined by evaluating the change in bioluminescence during the first week of treatment. The change in mean bioluminescence was lower in mice receiving silvestrol than in the control group (Figure 9A). A therapeutic response was observed in 36.4% of mice receiving 1.0 mg/kg body weight silvestrol and in 20% of mice receiving 0.4 mg/kg silvestrol, but only in 9% of mice in the control group (Figure 9B, C). The control group had a median survival of 28 days. Mice receiving either dose of silvestrol showed a significant increase in survival (p=0.024 for silvestrol 0.4 mg/kg vs. control, and p=0.037 for silvestrol 1.0 mg/kg vs. control). However, there was no significant difference in median survival in mice receiving 1.0 mg/kg body weight silvestrol (49 days) compared to those receiving 0.4 mg/kg silvestrol (42 days) (Figure 9D). We next performed multivariate analysis to determine predictive prognostic factors for survival. Multivariate analysis identified treatment with silvestrol and baseline bioluminescence as independent prognostic factors (Table 1). These data indicate that systemic administration of silvestrol was not only well tolerated in immunodeficient mice but resulted in significant improvement of the survival of tumor bearing mice.

**Table 1:** Predictive factors for survival.

Factors	Coefficient	Χ^2^	Hazard Ratio	95% Confidence Interval	*p*-value
**Treatment**		8.633			0.0133
**High dose**	-1.892	7.326	0.147	0.037-0.590	0.0068
**Low dose**	-1.371	5.266	0.247	0.075-0.816	0.0217
**Fibrosis (fibrotic liver)**	0.111	0.043	1.117	0.394-3.163	0.8351
**Luminescence > mean**	2.230	10.809	9.513	2.483-36.44	0.0010
**Body weight > mean**	-0.276	0.197	0.782	0.265-2.313	0.6570

Multivariate analysis, proportion hazard model; death

## Discussion

Hepatocellular cancer (HCC) is a globally prevalent malignancy for which effective therapies are needed. These studies identify that silvestrol is effective in inhibiting tumor growth in HCC cells both *in vitro* and *in vivo*, and thereby provide evidence of a novel therapeutic approach to these cancers. Silvestrol has been shown to inhibit translation, and therapeutic strategies targeting inhibition of protein translation are garnering attention as novel therapeutic agents for diverse human cancers. Thus, our results provide preclinical data that demonstrate the efficacy of targeting translation as a therapeutic strategy for HCC.

HCC frequently arises in the context of chronic hepatic injury resulting in hepatic fibrosis and cirrhosis [21]. The presence of hepatic fibrosis has the potential to impact tumor cell growth and therapeutic impact of anticancer agents [22,23]. In order to adequately mimic the tumoral microenvironment within which HCC occurs, we developed a disease relevant model of orthotopic HCC tumor cell xenografts in a fibrotic liver to examine the potential in vivo anti-tumor effects of silvestrol. An additional benefit of this model is the ability to examine the effect of anti-cancer therapies under both fibrotic and non-fibrotic conditions, thereby incorporating an assessment of tumor microenvironmental influences on tumor growth as well as anticancer effects. The impact of fibrosis on tumor latency and tumor growth was surprising. While we expected that fibrosis would enhance tumor formation, we were surprised to note that tumor growth was also modulated. Moreover, the differential effects on response to therapy should prompt re-evaluation of preclinical data that are derived from in vivo models that do not encompass the fibrotic milieu within which HCC arise.

Translation of most mRNAs can be regulated during the rate-limiting stage of initiation. Silvestrol can engage the eukaryotic initiation factor (eIF) 4A and inhibit the initiation of translation by depletion of eIF4A from the eIF4F complex [15,16]. Importantly, eIF4E is over-expressed in HCC [24]. When eIF4F activity is limiting, mRNAs with a short unstructured 5’-UTR can be translated whereas mRNA with long, G+C rich, highly structured 5’-UTRs are less efficiently translated because efficient ribosome loading is prevented by their complex structure [25]. Thus, the translation of highly structured, malignancy-related mRNAs is sensitive and dependent upon eIF4F for translation. A selective reduction in the translation of mRNAs that contain complex structured 5′ untranslated regions could result from exposure to silvestrol and potentially contribute to its therapeutic efficacy. Over-expression of eIF4E occurs in HCC and in transgenic models has been shown to promote HCC [24,26]. However, validation of this mechanism as a contributor of the anticancer effects of silvestrol would require selective modulation of eIF4A using other therapeutic strategies, and a systematic analysis of mRNAs that are deregulated by these interventions. Further analysis of selectively translated mRNAs modulated by silvestrol that encode proteins that are engaged in tumor cell survival and growth and tumor cell responses to the tumor microenvironment or to cellular stresses such as chemotherapy may be useful to understand specific pathways involved in HCC progression.

Recent studies have suggested that modulation of protein translation with depletion of survival factors with short half-life can enhance therapeutic responses. Our findings are consistent with these reports by indicating short term effects of silvestrol on Mcl-1 expression and cell apoptosis. These responses may contribute to the synergistic effects of silvestrol with two therapeutic agents that differ in their mechanisms of action, sorafenib and rapamycin. Sorafenib is an approved agent for HCC, and combination therapies with silvestrol are justified. Levels of eIF4F are regulated by mTOR, and mTOR pathways are an established target for cancer therapeutics and central to translational regulation. The use of rapamycin and other mTOR inhibitors is under active investigation for HCC and therapeutic effects of these agents may be enhanced by the concomitant use of silvestrol.

Importantly, silvestrol appears to be selective for neoplastic hepatocytes *in vivo*. Wild-type mice receiving silvestrol exhibited no overt hepatocellular damage after a 28-day course of 1.5 mg/kg given every other day. This observation is an important affirmation that silvestrol is unlikely to place a further toxic burden on the chemotherapy-tormented hepatic parenchyma of cancer patients, and thus should be a means of mitigating adverse events associated with multi-agent chemotherapy cocktails.

Silvestrol represents a structurally unique class of drugs with a novel mechanism that targets initiation of translation. Based on the antitumor effects of silvestrol in HCC, and its synergistic effects in combination with other therapeutic agents, we conclude that silvestrol has promise as an anticancer agent for HCC.
